# Measurement of the development level of tea tourism integration, spatiotemporal evolution and obstacle factors in China

**DOI:** 10.1371/journal.pone.0329974

**Published:** 2025-08-07

**Authors:** Zhendong Yang, Xiaoyu Wang, Jianwu Xiao

**Affiliations:** 1 School of Economics and Management, Central South University of Forestry and Technology, Changsha, China; 2 Research Center of Rural Revitalization and Green Development, Changsha, China; National Cheng Kung University, TAIWAN

## Abstract

Based on panel data from 18 major tea-producing provinces in China from 2013 to 2022, this study constructs an evaluation index system for tea tourism integration. it employs the entropy method, kernel density estimation, Markov chain, and obstacle model to analyze integration levels, spatiotemporal evolution patterns, and obstacle factors. The results show that: (1) China tea tourism integration shows an upward trend nationally, with four tea regions exhibiting a “Jiangnan> South China> Southwest> Jiangbei” developmental hierarchy, where the Southwest region demonstrates the fastest growth. (2) Interregional development disparities are widening, coupled with intraregional imbalances. Provincial development displays “club convergence” characteristics, with low-level areas exhibiting significant spatial spillover effects from neighboring high-level regions. (3) Common obstacle factors prevail across provinces, particularly industrial economic conversion efficiency, constituting the key constraint for integration development.

## 1. Introduction

As the birthplace of tea culture, China ranks first in the world in terms of tea cultivation scale and production [1]. As a traditional advantageous industry, the tea sector has played a vital role in promoting rural economic development, optimizing agricultural industrial structure, and improving regional ecological environments [2]. Meanwhile, tourism-owing to its strong multiplier effects, high degree of integration, and experiential consumption characteristics-serves as a core medium for activating the value of rural resources. On factors such as resource sharing, scene co-creation, and value co-production, tea and tourism possess an inherent basis for integration. At the same time, as an agricultural product rich in social and cultural attributes, tea’s development trajectory is closely linked with the tourism industry [3]. Each stage of the tea production chain can be continuously integrated with tourism’s six consumption elements, giving rise to new economic-consumption-oriented models, such as tea-garden experiences, cultural performances, and tea-related creative products [4].

However, due to lagging technological innovation on the production side, intensifying international competition, diversified consumer demand, and external constraints such as trade barriers, China’s tea production efficiency and value-added levels remain comparatively low, indicating considerable potential for economic improvement in the tea sector [5]. At the same time, the tourism industry continues to recover from the effects of the COVID-19 pandemic. Against this backdrop, tea tourism integration-as an innovative three-industry convergence system-demonstrates significant strategic prospects. The National Rural Industry Development Plan (2020–2025) emphasizes the key role of optimizing rural leisure tourism, stating that “expanding functions to drive format integration, and advancing the integration of agriculture with cultural, tourism, education, and health industries,” thus provides policy guidance and a developmental framework for tea tourism integration. Facing the dual pressures of declining returns from traditional tea cultivation and upgrading market demand, tea tourism integration can effectively revitalize both tea-garden ecological resources and tea-culture assets by transforming local production-end scenes and empowering cultural value on the consumption side of tourism. This process produces a virtuous cycle of “driving tourism with tea and driving tea with tourism,” thereby offering a sustainable development pathway for agricultural modernization and rural revitalization [6,7].

In academic circles, early research on the integration of tea tourism centered on theoretical definitions. Wang et al. [8] defined tea-cultural tourism as an immersive experience form centered on tea culture, emphasizing its cultural immersion features. Liu [9] further noted that the tea-industry chain and tourism-consumption chain reconstruct value through spatial superposition, thereby revealing the dynamic interactive nature of industry elements. Zhang et al. [10] proposed that tea tourism integration represents a deep fusion of the tea industry, tourism industry, and related service industries across three dimensions-formats, spatial configuration, and governance. At the practical level, scholars have broadened their perspectives to include development pathways and driving mechanisms, identifying a fusion logic driven endogenously by cultural ecology and externally by policy support and consumer upgrading [11–14]. Meanwhile, Gupta et al. [15] confirmed through cross-cultural comparison that participation in tea-art performances and the aesthetic value of tea-garden landscapes significantly influence visitor satisfaction, thereby providing empirical backing for customized tea tourism product design. Other researchers have systematically examined tea-tourist motivations [16], satisfaction [17], marketing management of tea tourism integration [18], and its social impacts and outcomes [19].

With the introduction of “coordination degree” and “industrial coupling” concepts [20], scholars began to adopt coupling coordination models-initially applied to regional economy-tourism-ecological environment system evaluations [21], and later extended them to evaluate tea tourism integration. Yi et al. [22] conducted the first tea tourism coupling coordination study in Zhejiang Province, constructing a model that used tea-industry and tourism-industry indicators to measure integration levels. Cheng et al. [23] developed a tea tourism-ecological environment indicator system to analyze coupling coordination in Fujian Province. Pang et al. [24] further expanded this model to 18 major tea-producing regions nationwide to quantify China’s tea tourism coupling degree. In addition, some scholars have employed grey relational analysis [25] and importance-performance analysis (IPA) [26,27] to measure tea tourism integration levels across different regions.

In summary, both domestic and international scholars have extensively explored tea tourism integration from theoretical and empirical perspectives. Theoretically, research has progressively built a comprehensive conceptual framework by defining tea-cultural tourism, elucidating industry-element interaction mechanisms, and proposing multidimensional integration models. Empirically, methods such as coupling coordination models, grey relational analysis, and IPA have been applied to measure regional integration levels from multiple angles. Nevertheless, existing research still exhibits the following shortcomings. First, coupling coordination models commonly used in the literature calculate coordination by separating industry indicators and often ignore intrinsic interdependencies among indicators. This approach biases the resulting coordination measures toward system-level assessments, without fully capturing the integration development level. Second, studies on the spatiotemporal dynamics and key constraints of tea tourism integration remain fragmented and lack systematic analysis.

Accordingly, this paper selects 18 tea-producing regions across China’s four major tea areas for the period 2013–2022. Guided by the “boundary reconfiguration-value co-creation” framework of industrial integration theory and the “core-edge” structure of regional development theory, we construct a comprehensive evaluation index system encompassing four dimensions:integration subjects, integration foundations, integration pathways, and integration efficiency. We employ the entropy method, kernel density estimation, Markov chain analysis, and obstacle degree modeling to explore tea tourism integration levels, their spatiotemporal evolutionary patterns, and primary barriers. This study enriches methodological approaches for measuring tea tourism integration, providing a practical, replicable framework. Moreover, as an empirical investigation grounded in a regionally distinctive Chinese industry, our analytical framework and research approach offer valuable insights for other countries or regions seeking to integrate agricultural and tourism industries, demonstrating international reference value and theoretical applicability.

## 2. Study area overview

Tea producing regions in China are mainly divided into Southwest Tea Region, Jiangnan Tea Region, South China Tea Region and Jiangbei Tea Region [28]. This geographic classification, universally recognized within China’s tea science community, reflects an integration of climate, topography, soil, and tea cultivar characteristics. These regions not only have long histories of tea cultivation and large-scale tea industries, but are also designated national or provincial key tea-producing areas. As such, they possess a solid foundation for tea tourism integration and benefit from strong policy support. At the same time, data availability in these regions is relatively high, enabling the construction of panel data models and time-series analyses. Therefore, this regional classification and sample selection are representative, scientifically sound, and feasible for capturing the overall status of tea tourism integration in China. Accordingly, this paper selects 18 tea-producing areas across China’s four major tea zones: the Southwest Tea Region (Yunnan, Guizhou, Sichuan, Chongqing), the Jiangnan Tea Region (Zhejiang, Anhui, Jiangsu, Hunan, Hubei, Jiangxi), the South China Tea Region (Guangdong, Guangxi, Fujian, Hainan), and the Jiangbei Tea Region (Gansu, Shaanxi, Henan, Shandong).

## 3. Research design and data sources

### 3.1 Construction of the indicator system

Based on principles of systematicity, scientific rigor, and data availability, and drawing on previous studies [22–24,28–33], we constructed a comprehensive evaluation index system for tea tourism integration development. This system comprises four first-level indicators: integration subjects, integration foundations, integration pathways, and integration efficiency. These are further broken down into fourteen second-level indicators-industrial entity, industry infrastructure, policy support, transportation provision, consumption level, supporting facilities, functional integration, talent integration, brand integration, cultural integration, livelihood improvements, consumption growth, industry-value enhancement, and economic advancement-and, ultimately, sixteen specific third-level indicators. These indices serve to measure the level of tea tourism integration; the detailed indicator list is presented in Table 1.

**Table 1:** Tea tourism integration development level evaluation index system.

First level indicators	Second level indicators	Third level indicators	Indicator calculation or description	Nature	Symbol
Integrating Entity	Industrial entity	Number of tea enterprises	\	+	X1
		Number of Tea Tourism Businesses as a Percentage	Number of tea tourism enterprises in the region/ Number of tea enterprises, %	+	X2
Foundation for Integration	Industrial foundation	Tea production	ton	+	X3
		Number of tourists	10,000 people	+	X4
	Policy support	Number of mentions of the tea tourism policy	Number of tea tours mentioned in government work reports, times	+	X5
	Transportation security	Road network density	Regional road mileage/land area, %	+	X6
	Consumption level	Per capita disposable income	yuan	+	X7
	Service	Percentage of restaurant and lodging industry above the limit	Restaurants and lodgings above the regional limit/national total restaurants and lodgings above the limit, %	+	X8
Integration Pathway	Functional integration	Level of development of tea tourism attractions	(Area of tea gardens/area of sown crops) x number of A-class scenic spots, %	+	X9
	Integration of talents	Number of higher education institutions	\	+	X10
	Brand fusion	Tea tourism branding level	Number of regional public brands for tea, pcs	+	X11
	Cultural fusion	Number of tea culture museums	\	+	X12
Integration Effect	Improve people’s livelihood	Value added per capita income in tea-producing areas	Disposable income per capita in the main tea-producing areas of the region – disposable income per capita in the same period of the previous year, yuan	+	X13
	Consumer growth	Value added of tourism consumption index	(Gross regional tourism receipts/total regional arrivals) – index for the same period of the previous year, %	+	X14
	Industrial efficiency	Value added of tea tourism industry	Value added of regional tea tourism industry, 100,000,000 yuan	+	X15
	Economic upliftment	Economic contribution of tea tourism industry	Regional tea tourism industry output value/ total regional GDP, %	+	X16

Integration subject dimension primarily reflects the resource supply and composition of market entities involved in tea tourism integration and serves as the basis for assessing integration potential, referred to as the industrial subject. The number of tea enterprises indicates the vibrancy of the industry and is a key measure of industrial scale and agglomeration. The proportion of tea tourism enterprises reflects the degree of organizational and operational convergence between the tea and tourism industries, directly illustrating the fusion of industrial boundaries.

Integration foundations encompass the external environment and support conditions that enable tea tourism integration and serve as a feasibility prerequisite.This dimension includes five second-level indicators: industry infrastructure, policy support, transportation provision, consumption capacity, and supporting facilities. Tea production directly reflects the resource supply capacity of the tea industry and constitutes the material foundation for tea tourism integration. The number of tourists measures regional tourism attractiveness. The number of mentions of the tea tourism policy indicates the government’s level of attention, directly influencing the integration process and mechanism design. Road network density determines the accessibility of tourist destinations, forming the basic condition for spatial integration and traffic flow conversion. Per capita disposable income of residents is a critical economic variable that measures regional consumption capacity and willingness to participate. The proportion of catering and accommodation businesses above a designated size reflects the development level of tourism services and serves as an important indicator of the integration of reception capacity and service support.

Integration pathways describe the development strategies, construction of carriers, and functional realization methods that drive tea tourism integration. This action process and structural mechanism comprises four second-level indicators: functional integration, talent integration, brand integration, and cultural integration. The development level of tea tourism attractions reflects the ability to integrate tourism resources and the depth of functional integration. The number of higher-education institutions supports industry innovation and sustainability in tea tourism integration. The level of tea tourism branding represents market influence and product recognition, indicating the visibility and competitiveness of tea tourism offerings in the consumer market. The number of tea culture museums serves as a spatial carrier for tea culture dissemination and visitor experience, acting as an important medium for achieving tea tourism integration.

Integration efficiency reflects the economic, social, and market feedback generated by tea tourism integration and represents the ultimate goal for measuring performance. This dimension covers four second-level indicators: livelihood improvements, consumption growth, industry-value enhancement, and economic advancement. Per capita income added value in tea-producing areas indicates whether tea tourism integration effectively promotes household income growth and rural revitalization, serving as a key indicator of social benefits and targeted impact. The increase in tourism consumption index measures market-level outcomes of tea tourism integration. The added value of the tea tourism industry represents the comprehensive output capacity of the integrated tea tourism sector and directly reflects the degree of deepening integration. Finally, the economic contribution of the tea tourism industry indicates its driving effect on regional economies and serves as a core indicator of the strategic value of tea tourism integration.

### 3.2 Modeling

#### 3.2.1 Tea tourism integration development level measurement model.

Based on the research method of Chen et al. [34], the entropy value method is used to calculate the score of tea and tourism integration development level. Based on the characteristics of information entropy, the information entropy of each indicator is calculated by eliminating the difference of the indicator’s magnitude through the standardization of the polarity processing to get the weight of each indicator in the evaluation and the comprehensive development level [35], and the specific steps are as follows.

Before the tea tourism integration development level measurement, due to the inconsistency of the index outline and unit, the data need to be standardized. In order to avoid the standardization of the results of the processing of meaninglessness, it is necessary to standardize the index data after the standardization of the standardization of the standardization of the index plus 0.01. The calculation formula is:


$${X^{\prime }}_{ij}=\frac{X_{ij}-X_{min}}{X_{max}-X_{min}}+0.01$$

Where $X_{ij}\;$denotes the jth raw data in column i,$\;{X^{\prime }}_{ij}$ denotes the normalized data, and $X_{\max}$and $X_{\min}$ denote the maximum and minimum values of the indicator, respectively.

Calculation of the weight of indicator j in year i $y_{ij}\backslash )$:


$$y_{ij}=\frac{{X\prime \prime }_{ij}}{\sum_{i=1}^{n}X\prime \prime ij}$$

Calculate the entropy value of the jth indicator $e_{j}\backslash )$:


$$e_{j}=-\frac{1}{\ln n}\sum_{i=1}^{n}y_{ij}\ln y_{ij}\mathrm{\backslash ]}$$

Calculation of the coefficient of variation for the jth indicator $g_{j}\backslash )$:


$$g_{j}=1-e_{j}\mathrm{\backslash ]}$$

Calculate the weight of the jth indicator $\omega_{j}\backslash )$:


$$\omega_{j}=\frac{g_{j}}{\sum_{j=1}^{p}g_{j}}\mathrm{\backslash ]}$$

Finally, Development Level of Tea Tourism Integration Index $U_{i}$ is calculated by multiplying the obtained weights $\omega_{j}$ with the standardized data ${X^{\prime }}_{ij}$, as shown in the formula below:


$$U_{i}=\sum_{j=1}^{p}\omega_{j}{X^{\prime }}_{ij}\mathrm{\backslash ]}$$

#### 3.2.2 Dynamic evolutionary models.

In statistics, kernel density estimation is a nonparametric probability density estimation method based on the weighting of the kernel function, whose core principle lies in revealing the characteristics of the spatial distribution of a random variable through local smoothing. The expression of the probability density function is:


$$f(x)=\frac{1}{Nh}\sum_{i=1}^{N}K\left(\frac{x-x_{i}}{h}\right)\mathrm{\backslash ]}$$

Where N is the number of selected samples, K(.) is the kernel density function, $x_{i}$ is the independent identically distributed samples, and h is the smoothing parameter of bandwidth. The article adopts the Gaussian kernel density function to explore the evolution law of tea tourism integration development, and its expression is as follows:


$$K(x)=\frac{1}{\sqrt{2\pi }}\exp\left(-\frac{x^{2}}{2}\right)\mathrm{\backslash ]}$$

#### 3.2.3 Spatial correlation measurement models.

The spatial correlation measure is mainly used to test the spatial correlation between regions and then reveal the regional synergistic evolution mechanism triggered by the integration process of tea tourism. In empirical analysis, spatial autocorrelation indicators are commonly used for quantitative assessment, and the most commonly used is Moran’s I, with the following formula:


$$I=\frac{\sum_{i=1}^{n}\sum_{j=1}^{n}W_{ij}\left(x_{i}-\dot{x}\right)\left(x_{j}-\dot{x}\right)}{S^{2}\sum_{i=1}^{n}\sum_{j=1}^{n}x_{ij}}\mathrm{\backslash ]}$$

Where,I is the Moran Index,$x_{i}$ denotes the development level of tea tourism integration in region i, $\dot{x}$ denotes the average value of the development water of tea tourism integration in all regions, $W_{ij}$ is the neighboring space weight matrix, and $S^{2}$ is the variance value of the sample.

#### 3.2.4 Characterization models of spatial and temporal shifts.

The temporal and spatial transfer characteristics of China’s tea tourism integration development level are investigated through Markov modeling. The traditional Markov chain is used to analyze the trend of the evolution of the development level of Chinese tea tourism integration over time by constructing the state transfer probability matrix [36], which has the following expression:


$$P_{ij}=\frac{n_{ij}}{n_{i}}\mathrm{\backslash ]}$$

Where $P_{ij}$ denotes the probability that the development level of tea tourism integration has changed from type i to type j, $n_{ij}$ is the number of regions that have changed from type i to type j, and $n_{i}$ denotes the total number of type i regions.

The spatial Markov chain is an extension method of the traditional Markov chain, the core of which is to decompose the traditional matrix for state transfer probabilities into multiple conditional transfer probability matrices by introducing a spatial weight matrix. Under the spatial lag type k condition, we quantify the transition probability of a region moving from state i in period t to state j in period t + 1, thereby revealing how spatial factors influence the upgrading of tea tourism integration development levels.

#### 3.2.5 Obstacle model.

The obstacle degree model can quantify the degree of influence of each evaluation index in the comprehensive evaluation and accurately screen out the key factors limiting the further development of things, so as to clarify the key obstacles, improve the accuracy of decision-making, and provide data support for the development of targeted optimization strategies. Therefore, using the obstacle degree model, the main obstacle factors affecting the tea industry and tourism industry are analyzed with the following formula [37].


$$O_{ij}=\frac{F_{j}I_{ij}}{\sum_{j=1}^{n}F_{j}I_{ij}}\mathrm{\backslash ]}$$

Where $O_{ij}$ is the barrier degree; $I_{ij}$ is the indicator deviation, expressed as the difference between 1 and the standardized value of a single indicator; and $F_{j}$ is the contribution of a single indicator, i.e., the weight of a single factor $\omega_{j}\backslash )$.

### 3.3 Data sources

Data from the China Statistical Yearbook, China Rural Statistical Yearbook, EPS database, provincial and municipal statistical yearbooks, China Agricultural Brand Research Center of Zhejiang University, enterprise data from Enterprise Search, and museum data from the China Museum Association. Very few missing data are supplemented by the moving average method and the linear interpolation method.

## 4. Results

### 4.1 Tea tourism integration development level measurement results and analysis

Based on the above methodology, the development level of tea tourism integration in 18 tea-producing regions in China was measured from 2013 to 2022, and the results are shown in Table 2.

**Table 2:** Measurement results of the development level of tea tourism integration in China, 2013-2022.

Region	Province	2013	2014	2015	2016	2017	2018	2019	2020	2021	2022
National	average	0.1410	0.1530	0.1687	0.1876	0.2063	0.2371	0.2662	0.2803	0.3087	0.3282
Southwest Tea Region	Yunnan	0.1784	0.1931	0.2049	0.2397	0.2945	0.3171	0.3699	0.4074	0.4697	0.4934
	Guizhou	0.1165	0.1299	0.1420	0.1905	0.2407	0.2871	0.3068	0.3237	0.3812	0.4013
	Sichuan	0.1589	0.1713	0.2112	0.2284	0.2391	0.2773	0.2999	0.3310	0.3587	0.3946
	Chongqing	0.0835	0.0897	0.1009	0.1122	0.1173	0.1489	0.1650	0.1662	0.1912	0.1954
Jiangnan Tea Region	Zhejiang	0.2215	0.2311	0.2679	0.3103	0.3395	0.3979	0.4487	0.4747	0.5034	0.5139
	Anhui	0.2166	0.2266	0.2404	0.2679	0.2784	0.3055	0.3405	0.3421	0.3704	0.3749
	Jiangsu	0.1649	0.1681	0.1746	0.1800	0.1844	0.2129	0.2215	0.2171	0.2530	0.2575
	Hunan	0.1474	0.1652	0.1946	0.2111	0.2557	0.2919	0.3492	0.3637	0.3892	0.4058
	Hubei	0.1781	0.2104	0.2174	0.2690	0.2777	0.2965	0.3197	0.3222	0.3577	0.3575
	Jiangxi	0.0690	0.0753	0.0875	0.0968	0.1292	0.1398	0.1544	0.1767	0.2003	0.2207
South China Tea Region	Guangdong	0.1659	0.1786	0.2038	0.2185	0.2531	0.2680	0.2882	0.3167	0.3467	0.3788
	Guangxi	0.0595	0.0659	0.0740	0.0793	0.1112	0.1393	0.1715	0.2016	0.2112	0.2541
	Fujian	0.2226	0.2398	0.2803	0.3025	0.3511	0.3973	0.4520	0.4477	0.5105	0.5531
	Hainan	0.1799	0.1963	0.1943	0.1580	0.1090	0.1824	0.2212	0.2347	0.2357	0.2781
Jiangbei Tea District	Gansu	0.0551	0.0584	0.0636	0.0671	0.0684	0.0733	0.0774	0.0766	0.1014	0.0968
	Shaanxi	0.1063	0.1139	0.1242	0.1355	0.1465	0.1592	0.1939	0.1948	0.1910	0.1985
	Henan	0.1275	0.1326	0.1426	0.1522	0.1586	0.1873	0.1996	0.2099	0.2419	0.2430
	Shandong	0.1367	0.1598	0.1686	0.2269	0.2344	0.2597	0.2916	0.3108	0.3179	0.3431

Based on the national average values across 18 provinces, the level of tea tourism integration increased from 0.1410 in 2013 to 0.3282 in 2022, corresponding to a compound annual growth rate of 9.82 percent and reflecting a robust upward trajectory. This trend mirrors the policy support afforded by recent rural revitalization and agricultural tourism integration initiatives enacted by central and local governments. Concurrently, widespread internet penetration and the application of big data technologies in rural tourism have improved the digital supply of tea tourism products and enabled precise marketing strategies, thereby sustaining consumer growth. During this period, Fujian Province consistently ranked first nationwide in tea tourism integration, reaching its peak in 2022 and exhibiting strong growth momentum. In contrast, Gansu Province registered a relatively low integration index of only 0.0968 in 2022, indicating substantial imbalance among provinces and significant interprovincial disparities.

**Fig 1:**
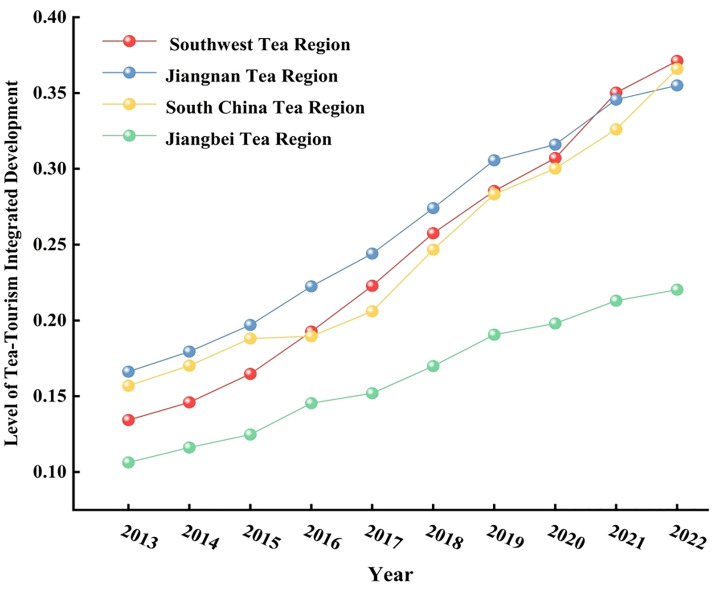
The average value of tea tourism integration development in the four major tea regions of China.

From the regional level (Fig 1), the level of development of tea tourism integration in all regions during the study period has increased year by year, but the interregional differences are significant. During this period, the average value of water in the Southwest Tea Region, South China Tea Region, South China Tea Region, and North China Tea Region was 0.2432, 0.2606, 0.2433, and 0.1637, respectively, presenting the pattern of “South China> South China> Southwest> North China,” but the regional development pattern is not the same. Specifically, the development level of tea tourism integration in the Southwest Tea Region jumped from the third in the region in 2013 to the first in 2022, with an average annual growth rate of 10.70%, which is the highest level in the four regions; and the Jiangnan Tea Region maintained the leading level in the region during the period from 2013 to 2020. However, from 2020 onwards, the growth rate of this tea region slows down significantly, falling to the third position in the region by 2022. Its average annual growth rate is 8.02%, the lowest in the region; South China Tea Region experienced a slowdown in growth during 2015−2017. However, since 2017, the growth rate of the region has continued to maintain a high level, with an average annual growth rate of 10.30%; Jiangbei Tea Region is at the lowest level in all of the inspection periods, but its trend of tea tourism integration and development is gradually accelerating, with an average annual growth rate of 8.54%. It is worth noting that during the 2019−2020 period, the level of tea tourism integration development in all regions showed a slowing down trend, which may be due to the economic impact of the sudden outbreak of COVID-19 at the end of 2019.

Overall, the Jiangnan Tea Region has maintained a leading position, owing to its comprehensive tea deep-processing industry chain and high-quality cultural tourism infrastructure. Its regional average integration level is markedly higher than that of other tea regions. However, as core provinces such as Zhejiang and Hunan have entered a mature phase of tea tourism integration, market saturation effects have emerged, slowing growth and making innovation-driven models the new source of expansion. By contrast, the South China Tea Region has leveraged the Greater Bay Area integration plan and “night economy” cultural-tourism pilot programs to enhance functional integration depth and brand influence through interprovincial tourism collaboration and brand promotion, rising to second place by 2022. The Southwest Tea Region benefited from national “dual circulation” and rural revitalization policies, with Yunnan and Guizhou receiving ample earmarked funding and policy support. By capitalizing on ethnic-minority cultural experiences and ecological tourism resources, they achieved rapid breakthroughs, ultimately securing first place. The Jiangbei Tea Region, however, lags behind due to limited transportation accessibility and low market visibility, resulting in a relatively slow integration pace.

### 4.2 Analysis of the temporal and spatial evolution of the development level of tea tourism integration

#### 4.2.1 Dynamic evolution analysis.

**Fig 2:**
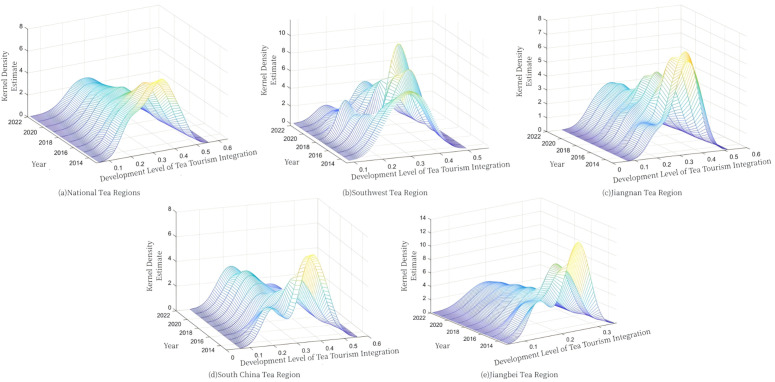
Dynamic evolution of the distribution of development level of tea tourism integration.

Using the Gaussian kernel density function of the kernel density estimation method, a three-dimensional kernel density distribution map of China and the four major tea regions in China was drawn by MATLAB software to visualize and analyze the dynamic evolution law of the development level of tea tourism integration in China, and the results are shown in Fig 2.

From the distribution location perspective, during the study period, the kernel density curves for China and all four tea regions generally shifted rightward, with the most pronounced shift occurring in the Southwest region. This finding indicates that integration levels in all tea regions have steadily improved, with the Southwest region exhibiting the fastest development rate, consistent with prior research.Examining distribution patterns, the national kernel density map shows that the main peak height gradually declined over time, reflecting a reduced concentration in integration levels and widening gaps between regions. In both the Jiangnan and Jiangbei regions, main peak heights also trended downward while peak widths narrowed, suggesting increased internal homogenization and balanced development within those regions, yet an overall lack of highly differentiated drivers. In the Southwest region, the main peak first rose and then fell, with the crest broadening; this indicates a clear agglomeration-diffusion process in which the Southwest’s integration advantages gradually spread to surrounding areas, narrowing intra-regional differences and ultimately flattening the distribution. Meanwhile, the South China region’s main peak initially fell and then rose while its width narrowed, displaying a distinct hierarchical structure; this suggests that high-level integration areas are emerging, the overall trend is shifting toward higher integration, but a pronounced gradient effect remains within the region, exacerbating inter-regional disparities. From the distribution of scalability, the four major tea regions have an obvious “left trailing” phenomenon, indicating that in the context of the overall enhancement, there are still some provinces with a lower level of tea tourism integration development, resulting in the development of the region not being balanced.

#### 4.2.2 Spatial correlation analysis.

Before conducting the Markov chain analysis, it is necessary to test whether there is spatial correlation in the development level of tea tourism integration between tea regions in China. The analysis was carried out using the Moran index, and the results are shown in Table 3.

**Table 3:** Spatial autocorrelation test for the development level of tea tourism integration in China.

Year	Moran’s I	P-value
2013	0.214	0.032
2014	0.227	0.028
2015	0.241	0.019
2016	0.268	0.007
2017	0.278	0.004
2018	0.305	0.001
2019	0.341	0.000
2020	0.342	0.000
2021	0.370	0.000
2022	0.375	0.000

The results show that during the examination period, the Moran indices of China’s tea tourism integration development level are all greater than 0, and none of the P-values is greater than 0.05, passing the significance test. Therefore, the development level of China’s tea tourism integration shows significant positive autocorrelation in space, and the correlation shows an upward trend.

#### 4.2.3 Characterization of temporal and spatial shifts.

Based on the previous spatial correlation study on the development level of tea tourism integration, Markov chain transfer probability matrix was introduced for analysis. The development level of tea tourism integration is categorized into Type I, Type II, Type III and Type IV, which represent low level areas (<25%), medium-low level areas (25% ~ 50%), medium-high level areas (50% ~ 75%) and high-level areas (>75%), respectively. The Markov chain state transfer matrix was constructed by MATLAB software to further analyze the spatio-temporal transfer characteristics of the development level of tea tourism integration in each province of China, and the results are shown in Table 4.

**Table 4:** Markov transfer probability matrix.

Spatial lag	T/(T + 1)	Ⅰ	II	III	IV	N
No lag	Ⅰ	0.7955	0.2045	0.0000	0.0000	44
	II	0.0233	0.6977	0.2791	0.0000	43
	III	0.0000	0.0000	0.7500	0.2500	40
	IV	0.0000	0.0000	0.0000	1.0000	35
Ⅰ	Ⅰ	0.8333	0.1667	0.0000	0.0000	12
	II	0.0000	0.7500	0.2500	0.0000	12
	III	0.0000	0.0000	1.0000	0.0000	4
	IV	0.0000	0.0000	0.0000	0.0000	0
II	Ⅰ	0.8667	0.1333	0.0000	0.0000	15
	II	0.0833	0.5833	0.3333	0.0000	12
	III	0.0000	0.0000	0.7727	0.2273	22
	IV	0.0000	0.0000	0.0000	1.0000	2
III	Ⅰ	0.7333	0.2667	0.0000	0.0000	15
	II	0.0000	0.8182	0.1818	0.0000	11
	III	0.0000	0.0000	0.3750	0.6250	8
	IV	0.0000	0.0000	0.0000	1.0000	26
IV	Ⅰ	0.5000	0.5000	0.0000	0.0000	2
	II	0.0000	0.6250	0.3750	0.0000	8
	III	0.0000	0.0000	1.0000	0.0000	6
	IV	0.0000	0.0000	0.0000	1.0000	7

From the no-lag section of Table 4, it is evident that diagonal probabilities significantly exceed off-diagonal values, indicating that tea tourism integration levels in Chinese provinces remain stable. Specifically, Class III and Class IV provinces have diagonal probabilities of 75 percent and 100 percent, respectively, compared with 79.55 percent and 69.77 percent for Class I and Class II. This suggests that high-level provinces are more likely to maintain their current status than low-level provinces, revealing a “club convergence” phenomenon in which strong provinces become stronger and weak provinces fall further behind. Moreover, examining values on either side of the diagonal shows that upward transition probabilities substantially exceed downward ones, and no “leapfrog” transitions occur between different tiers. In other words, provinces progress incrementally rather than jumping across integration levels.

When incorporating spatial lag into the traditional Markov chain transition matrix, the lagged results in Table 4 indicate three key findings. First, under spatial spillover effects, “rank locking” persists. Even with spatial lag, diagonal probabilities remain higher than off-diagonal values, confirming that provincial integration levels stay stable after accounting for geographic factors. Second, changes in provincial tea tourism integration levels do not occur in isolation but are influenced by spatial geographic context, exhibiting significant spillover effects. When a low-level province is adjacent to a high-level province, the probability of upgrading increases. For example, under a Class IV spatial lag, Class I and Class II provinces have 50 percent and 37.5 percent probabilities, respectively, of moving up one tier, both higher than the 26.67 percent and 18.18 percent probabilities observed under a Class III lag. This demonstrates that geographic proximity significantly affects integration outcomes, particularly for lower-level provinces. Third, adjacency to low-level provinces does not trigger downgrades, meaning that lagging regions do not impose negative effects on neighboring high-level provinces. When the spatial lag type is Class II, Class III and Class IV provinces each have a zero probability of downward transition, indicating that high-level provinces possess strong resilience and can maintain their integration advantages despite proximity to less developed neighbors.

### 4.3 Analysis of obstacles to the development level of tea tourism integration factors

Using the obstacle degree model, we calculated the mean obstacle degree for each of the sixteen specific indicators selected above, and analyzed the top five barriers by average value [38,39].

As shown in Tables 5 and 6, the obstacle factors for tea tourism integration exhibit clear commonalities across China’s provinces and tea regions, while numerical differences highlight the varying intensities of these constraints. Most notably, the economic contribution of the tea tourism industry (X16) ranks as the primary obstacle in all provinces and tea regions except Hainan, with mean obstacle degrees consistently exceeding 20 percent-for example, 21.77 percent in Yunnan, 26.28 percent in Zhejiang, 20.85 percent for the Southwest region, and 22.02 percent for the Jiangnan region. These high values quantitatively confirm that the core challenge in current tea tourism integration lies in inadequate industrial economic conversion, specifically manifesting as low industry value-added and homogeneous consumption scenarios that severely limit economic impact. Meanwhile, policy support (X5), level of development of tea tourism attractions (X9), and number of tea culture museums (X12) appear frequently among the top five obstacles in most provinces and tea regions. Although their obstacle degrees are lower than X16, they generally fall between 10 and 15 percent. For instance, X5 records 11.05 percent in Guizhou and 13.52 percent in Jiangsu; X9 reaches 13.77 percent in Anhui and 14.36 percent in Shandong; X12 is as high as 15.41 percent in Fujian. These values indicate that the precision of policy guidance, the maturity of attraction development, and the richness of cultural dissemination channels are critical factors affecting tea tourism integration, and therefore require urgent strengthening.

**Table 5:** Ranking of obstacle factors in 18 tea-producing provinces in China.

Region	Obstacle Factors				
	1	2	3	4	5
Yunnan	X16 (21.77)^a^	X9 (12.70)	X5 (10.68)	X2 (9.43)	X11 (8.36)
Guizhou	X16 (21.23)	X9 (10.56)	X5 (11.05)	X12 (12.72)	X1 (10.15)
Sichuan	X16 (21.34)	X12 (12.39)	X5 (11.72)	X9 (11.76)	X1 (9.14)
Chongqing	X16 (19.06)	X9 (12.43)	X5 (12.49)	X12 (12.69)	X3 (9.00)
Zhejiang	X16 (26.28)	X5 (13.12)	X1 (11.46)	X2 (10.57)	X12 (9.51)
Anhui	X16 (22.74)	X9 (13.77)	X5 (13.45)	X3 (9.33)	X1 (9.11)
Jiangsu	X16 (20.46)	X9 (13.49)	X5 (13.52)	X3 (10.31)	X1 (9.43)
Hunan	X16 (22.19)	X9 (14.10)	X5 (13.34)	X1 (10.09)	X3 (7.09)
Hubei	X16 (21.42)	X5 (13.16)	X9 (12.62)	X2 (9.27)	X3 (4.60)
Jiangxi	X16 (19.03)	X5 (11.98)	X9 (11.91)	X12 (12.08)	X3 (8.63)
Guangdong	X16 (21.40)	X9 (14.04)	X5 (13.68)	X3 (9.17)	X11 (8.42)
Guangxi	X16 (18.86)	X9 (12.02)	X5 (9.42)	X12 (12.73)	X3 (8.38)
Fujian	X16 (24.71)	X12 (15.41)	X5 (10.59)	X9 (9.90)	X2 (9.08)
Hainan	X5 (14.26)	X9 (13.80)	X12 (13.67)	X3 (10.50)	X1 (9.65)
Gansu	X16 (15.27)	X5 (12.80)	X9 (11.84)	X12 (11.81)	X3 (9.07)
Shaanxi	X16 (17.17)	X5 (13.28)	X12 (12.97)	X9 (11.22)	X3 (8.66)
Henan	X16 (18.70)	X9 (12.99)	X5 (13.13)	X3 (8.99)	X2 (8.53)
Shandong	X16 (21.79)	X9 (14.36)	X5 (14.84)	X3 (10.74)	X1 (7.71)

^a^Note: Figures in parentheses indicate the degree of impairment of the factor (%), same below.

**Table 6:** Ranking of obstacle factors in the four major tea-producing regions of China.

Regions	Obstacle Factors				
	1	2	3	4	5
Southwest Tea Region	X16 (20.85)	X9 (11.86)	X5 (11.49)	X12 (11.25)	X1 (9.07)
Jiangnan Tea Region	X16 (22.02)	X5 (13.10)	X9 (11.78)	X1 (9.46)	X12 (8.70)
South China Tea Region	X16 (17.12)	X12 (12.55)	X9 (12.44)	X5 (11.99)	X1 (7.82)
Jiangbei Tea District	X16 (18.23)	X5 (13.51)	X9 (12.60)	X12 (9.95)	X3 (9.37)

Despite the dominance of common obstacles, regional differences are apparent. Hainan is the sole exception: its chief obstacle is X5, with an obstacle degree of 14.26 percent, while X16 ranks only fourth (15.27 percent), reflecting Hainan’s unique geographic and economic structure. Nevertheless, X9 and X12 still record high obstacle degrees of 13.80 percent and 13.67 percent, respectively, in Hainan-indicating that policy implementation, attraction development, and cultural dissemination remain pressing constraints. Moreover, although the ranking of key obstacles (X16, X5, X9, X12) aligns closely with area patterns, the specific values and order of secondary obstacles (for example, X1 or X3 exceeding 10 percent in Zhejiang and Shandong) exhibit subtle variations. This suggests that, while addressing the core constraint of “insufficient economic conversion,” provinces must also tailor strategies to their own identified weaknesses, as reflected by their obstacle degrees, to effectively remove obstacles to tea tourism integration.

## 5. Discussion

Tea tourism integration, as a key pathway for promoting comprehensive rural revitalization and achieving sustainable regional economic development, has evolved academically from theoretical discussions to empirical analysis. In most empirical studies, scholars measure integration levels by constructing discrete indicator systems and employing coupling coordination models. For example, Yi et al. [22] developed a coupling coordination model by building separate indicator sets for the tea and tourism industries, while Cheng et al. [23] constructed a three-dimensional framework encompassing the tea industry, tourism industry, and ecological environment indicators to calculate coupling coordination degrees. However, these studies often fail to fully account for the intrinsic interdependencies among indicators, and their measurements focus primarily on coordination rather than true integration levels. Moreover, existing research on China’s tea tourism integration generally adopts a single perspective and lacks an in-depth analysis of spatiotemporal dynamics and key obstacle factors. Building on industrial integration theory and the existing literature, this study advances the empirical investigation of tea tourism integration. By employing multiple methods-namely, the entropy method, kernel density estimation, Markov chain analysis, and obstacle degree modeling-this paper constructs a comprehensive evaluation index system to measure integration levels, examines China’s spatiotemporal evolution of tea tourism integration, and identifies primary obstacles. Compared to previous work, our approach broadens the research perspective and overcomes the limitations of traditional coupling coordination models, thus providing a theoretical foundation for accurately measuring integration levels and formulating scientifically grounded integration policies.

Our findings reveal that, from 2013 to 2022, China’s average tea tourism integration level rose from 0.1410 to 0.3282, indicating steady overall progress. Viewed from the provincial distribution, higher integration levels are concentrated in the southeastern coastal and southwestern regions. Notably, Lin et al. [13] reported a “high in the central region, low at both ends” spatial pattern for coupling coordination degrees, a finding that diverges from ours-likely due to differences in indicator selection and measurement methods between the two studies. From the four-region perspective, Jiangnan, South China, and Southwest perform similarly, whereas the Jiangbei region clearly lags behind, consistent with Pang et al. [24]. This disparity stems from unsuitable climatic conditions in parts of northern China and limited tourism and economic resources in some provinces, which weaken the foundational conditions for integration. It is therefore advisable to implement differentiated regional strategies [40]. For Jiangnan, South China, and Southwest, policies should emphasize “improving quality, strengthening collaboration, and guiding innovation” by extending and deepening the tea tourism industry chain to boost integration depth and conversion efficiency. For Jiangbei, efforts must focus on improving foundational conditions such as transportation accessibility, public services, and resource introduction to solidify the integration base.

The study also finds that despite the year-by-year rise in overall integration levels, intraregional imbalances remain pronounced. Spatiotemporal analysis reveals a coexistence of “club convergence” and “gradient diffusion.” Kernel density estimation shows national integration concentration has decreased while regional gaps have widened; the Markov chain analysis further demonstrates that high-level provinces have a significantly greater probability of maintaining their status than low-level provinces, creating a “rich get richer” effect. This indicates that tea tourism integration in China exhibits a spatial pattern of “agglomeration advantage expansion,” consistent with the “Matthew effect” in regional development theory. In addition, we observe significant positive spatial spillover effects across provinces, indicating that geographic proximity-particularly for low-level provinces-plays an important role in shaping integration processes. This finding enriches our understanding of uneven regional integration mechanisms and addresses gaps in Wei et al.’s [30] study of spatial dynamics. Accordingly, future policy design at national or provincial levels should strengthen regional linkage mechanisms-through fiscal transfers, cross-regional brand co-development, and benefit-sharing arrangements [41]—to guide “strong regions to lead weak regions” and achieve balanced development of integration resources.

Furthermore, our obstacle factor analysis indicates that inadequate industrial economic conversion is the core bottleneck limiting deeper integration. This finding suggests that current tea tourism integration models still reside at an “industrial stacking” stage and have not achieved deep value-chain integration, as evidenced by low value-added and homogeneous consumption scenarios. This observation echoes Liu et al.’s [9] noted phenomenon in early integration stages of “overemphasis on form over substance” and “focus on scale at the expense of efficiency.” However, by systematically diagnosing obstacles, we further quantify and confirm that insufficient economic conversion efficiency is the paramount barrier, providing a basis for targeted interventions. To move toward high-quality development, integration must shift from “surface coordination” to “deep integration” by constructing complete industry chains, strengthening cultural expression and product innovation, and enhancing digitalization and smart tourism capabilities [42] to achieve high-level synergy among industry elements, service functions, and spatial forms.

This study has certain limitations, which also offer directions for future research. First, our analysis relies primarily on provincial-level panel data and does not drill down to county or core scenic area levels; subsequent studies could incorporate field survey data to further explore the mechanisms driving integration and measure levels at finer scales. Second, due to data availability constraints, some potential indicators were not included; future research can further refine and expand the evaluation index system for tea tourism integration.

## 6. Conclusions

The article constructed a comprehensive evaluation index system for the development level of tea tourism integration based on four aspects: integration subject, integration foundation, integration path and integration efficiency, and measured the development level of tea tourism integration in 18 tea-producing provinces of China’s four major tea regions. We also analyzed the temporal and spatial dynamic evolution of China’s tea tourism integration development level and obstacle factors by using the kernel density estimation method, Moran index, Markov chain, and obstacle degree model. The following conclusions were drawn: First, the development level of tea tourism integration in China during the period of 2013–2022 shows an overall upward trend. From the perspective of the four major tea regions, the average value of development water during the study period shows the pattern of “Jiangnan> South China> Southwest China> Jiangbei,” in which the development level of tea tourism integration in the Southwest China Tea Region jumped from the third to the first in the region, and the average annual growth rate is the highest level in the four major regions. Secondly, China’s tea tourism integration development level of concentration has been reduced, the difference in development level between the provinces is expanding, the four major tea regions within the lower level of development of provinces, inter-regional and intra-regional imbalance still exists. The probability of maintaining the current state of high-level provinces is higher than that of low-level provinces, indicating that there is a “club convergence” phenomenon in the development level of tea tourism integration in China’s tea-producing provinces, and there is no “leapfrog” development. At the same time, the spatial geographic environment of tea tourism integration development level of dynamic transfer has a significant impact, when the low-level provinces neighbor high-level provinces, the probability of its transfer to the higher level is higher, reflecting the obvious spatial spillover effect; and the existence of low-level provinces does not have a negative effect on the development of high-level provinces. Thirdly, the “industrial economic transformation efficiency” is the core obstacle to the integration of tea tourism, reflecting the low value-added of the tea tourism industry chain and the homogenization of the consumption scene. At the same time, provinces need to strengthen policy guidance, scenic area construction, and tea culture dissemination to promote the sustainable development of tea tourism integration in China.
